# A Practical Philanthropist

**Published:** 1890-12

**Authors:** 


### HALL'S
### JOURNAL OF HEALTH

TRUTH DEMANDS NO SACRIFICE; ERROR CAN MAKE NONE.

Vol. 37. DECEMBER, 1890. No. 12.

### A PRACTICAL PHILANTHROPIST.

In 1792, among the rugged mountains of Virginia, Samuel Miller first saw the light. His early life was a continual struggle against the many obstacles which stood in the way of his determination to make something of himself, despite the discouraging nature of his surroundings. In order to acquire a tolerable education, he sacrificed many comforts which, at the best, were scanty enough. His first employment worth mentioning was that of school teacher in a region where schools were uncommon and school committes none too critical.

At length, turning his attention to trade, he succeeded so well that he eventually found himself the possessor of a considerable fortune, but he never forgot his humble beginnings nor the means of his prosperity, and, looking back upon the hard road over which he had been compelled to pass, he resolved to clear it of some of its forbidding obstacles, that those who should come after him might find the way easier in the pursuit of a fitting education and employment. Besides other charities, he established the Miller Manual Labor School at Albermarle, Virginia, and endowed it with a fund of over a million dollars. The pupils are of both sexes ; the children of poor parents and orphans without the means to provide for themselves, whose ages range from ten to eighteen, to whom, for the time being, every thing is free.

The institution is anti-sectarian, and there, not only are the educational advantages ample, but the manual training embraces a wide range of mechanics and the useful arts adapted to the requirements of both sexes in relation to their ability and prospects, so that at their graduation day they may enter the world's civil ranks fully equipped to hold their place with the foremost.

It is now scarcely a decade since this worthy charity was set on foot by the wisdom and benevolence of its founder, who had been made to feel its necessity in the early years of his own struggles out of the poverty and ignorance which hedged him in as a wall. Now the school numbers nearly three hundred pupils, of whom one-third are girls.

In the wide world we know of a no more beneficent accomplishment, and the name of Samuel Miller deserves to be enrolled among the worthiest benefactors of his race.

